# The Efficacy and Safety of Remimazolam Tosilate Versus Dexmedetomidine in Outpatients Undergoing Flexible Bronchoscopy: A Prospective, Randomized, Blind, Non-Inferiority Trial

**DOI:** 10.3389/fphar.2022.902065

**Published:** 2022-06-02

**Authors:** Xingfang Chen, Deqian Xin, Guangjun Xu, Jing Zhao, Qing Lv

**Affiliations:** ^1^ Department of Anaesthesiology, Liaocheng People’s Hospital, Liaocheng, China; ^2^ Department of Anesthesiology, Yantai Yuhuangding Hospital, Yantai, China

**Keywords:** remimazolam tosilate, dexmedetomidine, outpatients, flexible bronchoscopy, remifentanil

## Abstract

**Purpose:** This study aimed to compare the efficacy and safety of remimazolam tosilate-remifentanil (RT-RF) vs dexmedetomidine-remifentanil (Dex-RF) for outpatients undergoing fiberoptic bronchoscopy (FB).

**Patients and methods:** We conducted a double-blind, randomized, prospective study involving a total of 146 outpatients undergoing FB divided into two groups. The RT-RF (RR) group (*n* = 73) received an initial dose of 12 mg/kg/h of RT for 10 min followed by a maintenance dose of 1–2 mg/kg/h, while the Dex-RF (DR) group (*n* = 73) received an initial dose of 0.5 μg/kg of Dex for 10 min followed by a maintenance dose of 0.2–0.7 μg/kg/h. All outpatients also received 0.05–0.2 μg/kg/min RF to maintain the Modified Observer’s Assessment of Alertness and Sedation (MOAA/S) scale <3. The primary outcome was rate of successful FB completed. Secondary outcomes were time metrics, hemodynamics, intubating conditions, oxygen saturation, coughing severity, number of remedies, total dose of fentanyl, RF, RT, and Dex, incidence of dreaming, patient and bronchoscopist satisfaction, willingness to repeat bronchoscopy, and adverse events.

**Results:** The FB successful completion rate was 94.52% (95% CI: 89.20–99.90) in the RR group and 91.78% (95% CI: 85.30–98.20) in the DR group. Compared with patients in the DR group, the onset time, time to fully alert, and hospital discharge were all significantly shorter in the RR group (*p* < 0.01), and hemodynamics were more stable in the RR group. Intubating conditions, clinically acceptable intubating conditions, lowest oxygen saturation, coughing severity, consumption of fentanyl and RF, number of remedies, and patient and bronchoscopist satisfaction were similar between the groups (*p* > 0.05), as were demographic characteristics, incidence of dreaming, willingness to repeat bronchoscopy, and adverse events (*p* > 0.05).

**Conclusion:** RT-RF has non-inferior efficacy, better time metrics and hemodynamic stability for outpatients undergoing FB than Dex-RF.

**Systematic Review Registration**: [http://www.chictr.org.cn/showproj.aspx?proj=66673], identifier [ChiCTR2000041524].

## Introduction

Flexible bronchoscopy (FB), a procedure commonly used for the diagnosis and management of respiratory diseases, has experienced a remarkable increase in both number and complexity since its first application in 1968 (Mouritsen et al., 2020; Strohleit et al., 2021). FB is, however, an invasive procedure, and to relieve the patient’s anxiety, pain, stress, coughing, and hemodynamic fluctuations, as well as to facilitate the procedure, FB is usually performed under moderate to deep sedation, according to the complexity and expected duration, without compromising hemodynamics and oxygenation. Neuromuscular blocking agents (NMBAs) are utilized unless contraindicated (Minami et al., 2021; Tao et al., 2022). However, there is still no standardized sedation protocol available for outpatients undergoing FB despite the rapid development of newer drugs (ie remifentanil (RF), dexmedetomidine (Dex), and remimazolam) and equipment (ie supraglottic airways and mechanical jet ventilators). (de Lima et al., 2018; Maurel et al., 2020).

Total intravenous anesthesia (TIVA) is commonly used during FB; however, each drug used has its limitations (Purugganan, 2008). Midazolam, which acts on the inhibitory transmitter γ-aminobutyric acid (GABA) receptor in the ascending reticular activating system, remains one of the most commonly used sedatives during FB due to its favorable pharmacokinetic and pharmacodynamic properties. In addition, it can be rapidly counteracted by flumazenil. However, it is characterized by significant variability in metabolic clearance, and resedation may occur due to the cumulative effects of its active metabolites and numerous drug–drug interactions, particularly in elderly patients (Lee et al., 2019). Propofol is usually used in combination with benzodiazepines or opioids because of the increased risk of oversedation, airway obstruction, pain on injection, allergic reaction from egg and soybean components, and propofol infusion syndrome (Zha et al., 2021). Dex is a highly selective α2 adrenergic agonist with sedative and analgesic properties that can preserve airway reflexes, expand the smooth muscle of the trachea, and inhibit the cough response without causing respiratory depression. However, it may cause bradycardia and hypotension and has a slow onset when used separately (Ryu et al., 2012). Ketamine is a potent bronchodilator that can preserve airway patency and respiratory function, and has with sympathomimetic properties. However, ketamine may cause delirium, hallucinations, higher incidence of intense cough, and hypersalivation (Bhat et al., 2019). In summary, the ideal sedatives should be characterized by rapid onset, short action, wide margin of safety, minimal residual sedation, and minimal side effects; in addition, they should be rapidly reversible.

Remimazolam tosilate (RT) is an ester-based benzodiazepine that can be rapidly hydrolyzed into inactive metabolites by ubiquitous tissue esterases other than cytochrome-dependent hepatic pathways. The onset of action of remimazolam is 1–3 min and metabolic half-life is 0.75 h; in addition, its action can be completely antagonized by fumazenil. As a result, remimazolam is classified as a “soft drug.” (Wesolowski et al., 2016) Previous studies reported that compared to midazolam, remimazolam increased the rate of procedure success, registered superior restoration of neuropsychiatric function, and decreased the rescue medication and time to recovery during procedural sedation (Kilpatrick, 2021; Lee and Shirley, 2021). The aim of our study was to compare the safety and efficacy of remimazolam tosilate-remifentanil (RT-RF) with dexmedetomidine-remifentanil (Dex-RF) for outpatients undergoing FB.

## Material and Methods

### Patients

This single-center, prospective, double-blind, randomized, trial was performed from December 2020 to November 2021 at Liaocheng People’s Hospital. The protocol was approved by our center’s institutional review board (No. 2020045). Written informed consent was obtained from all patients or their legally authorized representatives before the start of the procedure. This trial is an extension of our initial protocol which is registered in the Chinese Clinical Trial Registry (ChiCTR2000041524).

A total of 187 outpatients who underwent FB from December 2020 to November 2021 were recruited in our hospital. Inclusion criteria were as follows: outpatients undergoing FB with moderate sedation; aged 45–65 years; American Society of Anesthesiologists (ASA) grade I-II; procedure time ≤30 min; and oxygen saturation (SpO_2_) > 90% in room air. Exclusion criteria were as follows: history of known allergy to local anesthetics (RF, Dex, midazolam, and propofol or RT); pre-existing lung disease (such as chronic obstructive pulmonary disease (COPD), respiratory failure, and asthma); severe sleep apnea syndrome (apnea-hypopnea index >40); body mass index (BMI) > 30 kg/m^2^; communication barrier; history of neuropsychiatric disorder, cerebrovascular disease, or abnormalities of renal or hepatic function; nasopharyngeal surgery; second- or third-degree atrioventricular block; bradycardia (heart rate (HR) < 60 beats per minute); drugs or alcohol abuse; and participating in other clinical trials less than 3 months prior.

### Randomization and Blinding

An anesthesiologist who was not involved in the trials performed randomization using sealed envelopes. This anesthesiologist also signed the informed consent, prepared the drug, and assessed the outcomes. All procedures were performed by the same bronchoscopist and anesthesiologist group, all of whom were blind to the group assignment. All patients were also unaware of the allocation. Group allocation results were unblinded after the end of study.

### Anesthetic Management

All patients were fasted for 8 h from solids and 2 h from clear fluids before FB according to the international multidisciplinary consensus statement (Green et al., 2020). No premedication was administered. An intravenous cannula was inserted into the right forearm vein. Standard hemodynamic monitoring included electrocardiogram (ECG), HR, noninvasive blood pressure (NIBP), SpO_2_, end-tidal carbon dioxide (PetCO_2_), and respiratory rate (RespR). After 10 min of airway nebulization with 10 ml of 1% lidocaine, all patients received 100% high-flow oxygen through a face mask for 5 min before the procedure. During the procedure, fentanyl 1 μg/kg and dexamethasone 0.1 mg/kg were injected intravenously at the same time and O_2_ at 4 L/min was applied via nasal cannula. Hemodynamics were recorded every 3 min until hospital discharge.

Anesthesia induction was performed with RT 12 mg/kg/h or Dex 0.5 μg/kg for 10 min. Anesthesia was maintained using RT 1–2 mg/kg/h or Dex 0.2–0.7 μg/kg/h in combination with RF 0.05–0.2 μg/kg/min to keep the Modified Observer’s Assessment of Alertness and Sedation (MOAA/S) scale <3 at the discretion of the anesthesiologist (Pastis et al., 2022). Five milliliters of 1% lidocaine were sprayed over the vocal cords, trachea, and right and left main bronchi through the bronchoscope channel for cough suppression. Supplemental lidocaine was given at the discretion of the bronchoscopist if cough interfered with the procedure and the total dose of lidocaine never exceeded 5 mg/kg. At the end of the procedure, all patients were routinely treated with 0.2 mg flumazenil to reduce the aftereffects of RT and transferred to the recovery room. The modified Brice questionnaire was used to evaluate the incidence of dreaming, and was administered to patients before discharge from the recovery room (Chen L et al., 2021).

In cases of insufficient sedation during FB, patients received a maximum of three doses of midazolam in any 12-min window at intervals of ≥2 min at the discretion of the anesthesiologist. In addition, the use of fentanyl 25 ug was permitted in every case, with a maximum of 200 ug administered until adequate analgesia. If sedation was still insufficient, treatment failure was declared, and 10–30 mg propofol was the only rescue sedative medication used.

### Outcome Measures

The primary outcome was the rate of procedures completed successfully. Secondary outcomes included the following: demographic characteristics; time metrics; hemodynamic measurements; intubating conditions (combining three variables: conditions of inserting the rigid bronchoscope, vocal cord position, and cough occurrence); lowest oxygen saturation; severity of coughing; number of remedies of midazolam, propofol, lidocaine, and vasoactive drugs; total dose of fentanyl, RF, RT, and Dex; incidence of dreaming (modified Brice questionnaire: 5-point Likert scale); patient and bronchoscopist satisfaction; willing to repeat the bronchoscopy; and adverse events (evaluated on the basis of the National Cancer Institute Common Terminology Criteria for Adverse Events ver. 4.0).

Time metrics were defined as follows: onset time (from injection of the sedative drug to the start of FB); procedure time (from the start to the end of FB); time to fully alert (from completion of FB to patients reaching an MOAA/S score of 5); and time to hospital discharge. Hemodynamic measurements were recorded at the following time points: arrival at the examination room (T1), immediately before start of FB (T2), 3 min after start of FB (T3), 6 min after start of FB (T4), 9 min after start of FB (T5), end of FB (T6), 3 min after FB (T7), 6 min after FB (T8), 9 min after FB (T9), and at hospital discharge (T10). The severity of coughing was graded based on the number of episodes of cough as follows: grade 0 (severe), ≥5 coughs; grade 1 (moderate), 3–4 coughs; grade 2 (minimal), 1–2 coughs; grade 3 no coughing.

Hypoxia was defined according to previous studies, as oxygen desaturation (SpO_2_ 75–89% for ≤60 s) and severe oxygen desaturation (SpO_2_ < 75% at any time or <90% for >60 s) (Zha et al., 2021). In the presence of hypoxia, the following maneuvers were carried out as required: oxygen delivery increased to 10 L/min, verbal and tactile stimulation, chin lift, jaw thrust, bronchoscope removal, face mask and manual ventilation, and tracheal intubation for mechanical ventilation. Respiratory depression was defined as < 8 breaths per minute. Hypotension was defined as mean blood pressure (MBP) decreased >20% compared with baseline and was treated with 40 μg phenylephrine or 6 mg ephedrine. Hypertension was defined as MBP increased >20% compared with baseline and was treated with 10–15 mg urapidil. Bradycardia was defined as HR < 60 beats per minute or reduction >20% compared with that at baseline and was treated with 0.2–0.4 mg atropine.

### Statistical Analysis

The sample size calculation was based on the results from our preliminary study. We assumed that the success rates of Dex and RT sedation would both be 80%. The predefined non-inferiority margin was an absolute difference of 15% between groups for the primary endpoint. With a non-inferiority margin of 20% on the relative scale, a power of 80%, and a one-sided alpha of 2.5%, the total sample size needed was 126. Assuming a dropout rate of 15%, a minimum of 73 patients were recruited for each group.

The distribution and homogeneity of the data were checked using the Shapiro–Wilk and Levene tests. Continuous outcomes were presented as means ± standard deviations (SDs) or medians and interquartile ranges, and analyzed with the Student’s t-test or Kolmogorov–Smirnov Z-test as appropriate in terms of data distribution. Repeated-measures analysis of variance was used with respect to hemodynamic measurements between the two groups. Qualitative data are presented as numbers and frequencies. Between-groups comparisons of qualitative variables were analyzed using χ2 of Fisher’s exact tests. *p* values of <0.05 were considered statistically significant. Statistical analysis was performed using SPSS software 24.0 (SPSS Inc., Chicago, IL, United States).

## Results

### Patient Demographic Characteristics

**FIGURE 1:**
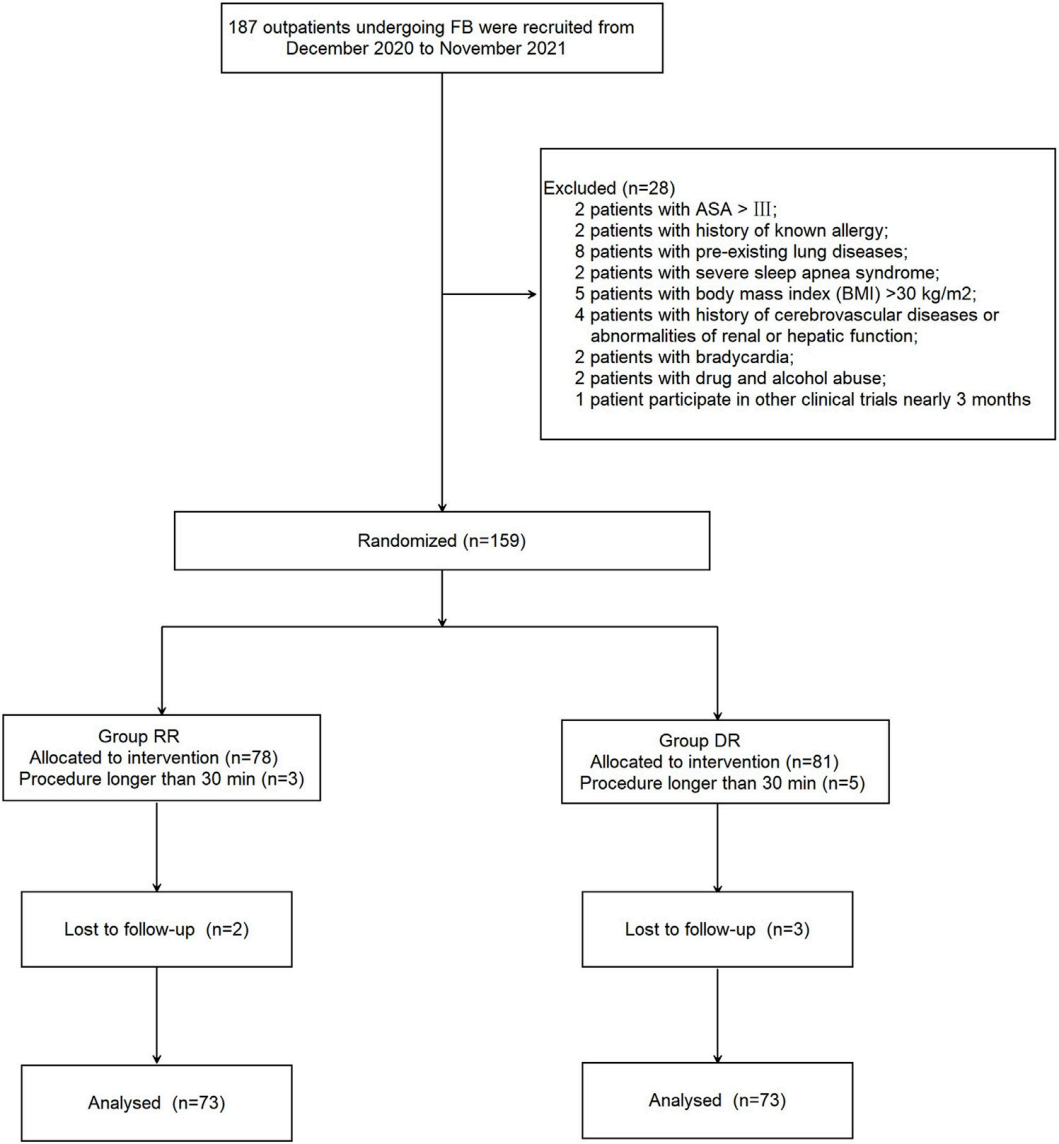
Patient flowchart with CONSORT guidelines.

A total of 28 outpatients were excluded from this study for the following reasons: ASA > III (*n* = 2); history of known allergy to propofol (*n* = 2); chronic obstructive pulmonary disease or asthma (*n* = 8); severe sleep apnea syndrome (*n* = 2); BMI >30 kg/m^2^ (*n* = 5); history of cerebrovascular diseases or abnormalities of renal or hepatic function (*n* = 4); bradycardia (*n* = 2); drugs or alcohol abuse (*n* = 2); and participated in other clinical trials less than 3 months prior (*n* = 1). In addition, 13 patients were excluded because either the procedure took longer than 30 min (*n* = 8; 3 patients in the RR group and 5 patients in the DR group) or patients were lost to follow-up (*n* = 5; 2 patients in the RR group and 3 patients in the DR group). As a result, 146 patients were randomized into two groups (*n* = 73, each; Figure 1); patients in the RT-RF (RR) group received an initial dose of RT 12 mg/kg/h for 10 min followed by a maintenance dose of 1–2 mg/kg/h, whereas patients in the Dex-RF (DR) group received an initial dose of 0.5 μg/kg of Dex for 10 min followed by a maintenance dose of 0.2–0.7 μg/kg/h. Patients in both groups also received 0.05–0.2 μg/kg/min of RF to maintain the Modified Observer’s Assessment of Alertness and Sedation (MOAA/S) scale <3. There were no significant differences between the two groups with respect to patients’ demographic characteristics and perioperative date (*p* > 0.05; Table 1).

**TABLE 1:** Demographic characteristics and perioperative data.

Variable	Group RR (*n* = 73)	Group DR (*n* = 73)	*p*-Value
Age (years)	57.05 ± 5.60	56.05 ± 5.99	0.299
Sex (male/female)	55/18	53/20	0.706
History of smoking, n (%)	48 (65.75%)	47 (64.38%)	0.862
FEV1/FVC (%)	90.36 ± 3.11	89.65 ± 3.13	0.171
Height (cm)	167.67 ± 5.01	166.56 ± 5.87	0.221
Body weight (kg)	68.97 ± 5.33	69.64 ± 6.20	0.484
BMI (kg/m^2^)	24.58 ± 2.19	25.11 ± 2.02	0.126
ASA I/Ⅱ (n)	25/48	27/46	0.730
Comorbidity, n (%)			0.862
Hypertension	22 (30.14%)	19 (26.03%)	
Diabetes	9 (12.33%)	12 (16.44%)	
Coronary heart disease	13 (17.81%)	6 (8.22%)	
Indication, n (%)			0.974
Lung cancer	23 (31.51%)	24 (32.88%)	
Pneumonia	25 (34.25%)	27 (36.99%)	
Hemoptysis	9 (12.33%)	10 (13.70%)	
Pulmonary tuberculosis	4 (5.48%)	4 (5.48%)	
Interstitial lung disease	6 (8.22%)	4 (5.48%)	
Others	6 (8.22%)	4 (5.48%)	
Procedure, n (%)			0.813
Endobronchial inspection	28 (38.36%)	27 (36.99%)	
Bronchoscopic biopsy	33 (45.21%)	31 (42.47%)	
Bronchoalveolar lavage	12 (16.44%)	15 (20.55%)	

Variables presented as mean ± SD, or number of patients n (%). FEV1 = forced expiratory volume in the first second; FVC , forced vital capacity; BMI , body mass index; ASA , american society of anesthesiology.

### Efficacy Outcomes

The successful completion rate of the FB procedure was 94.52% (69/73; 95% confidence interval (CI): 89.20%–99.90%) in the RR group and 91.78% (67/73; 95% CI: 885.30%–98.20%) in the DR group. The difference in the successful completion rate of the procedure between the two groups was 2.74% (95% CI: 1.70%–3.90%). As a result, RT-RF was considered non-inferior to DEX-RF in outpatients undergoing FB because the higher limit of the 95% CI for the difference in the successful completion rate of the procedure was not greater than the non-inferiority limit of 20% (Table 2).

**TABLE 2:** Difference of the successful completion rate of procedure between two groups.

Variable	Group RR (*n* = 73)	Group DR (*n* = 73)	*p*-Value
Procedure success, n (%)	69 (94.52%)	67 (91.78%)	0.745
95% CI	(89.20%, 99.90%)	(85.30%, 98.20%)	
Difference in rates	2.74%		
95% CI	(1.70%, 3.90%)		

Variables presented as number of patients n (%). CI , confidence interval.

Compared with patients in the DR group, patients in the RR group had a significantly shorter onset time (13.22 ± 1.70 min vs 15.12 ± 2.07 min), time to fully alert (2.52 ± 1.11 min vs 3.62 ± 1.28 min), and hospital discharge (18.58 ± 2.98 min vs 21.21 ± 3.60 min) (*p* < 0.01; Table 3). However, there was no significant difference with respect to procedure time (18.12 ± 3.32 vs 18.42 ± 3.00) between the two groups (*p* = 0.566, Table 3).

**TABLE 3:** Difference of second outcomes between the two groups.

Variable	Group RR (*n* = 73)	Group DR (*n* = 73)	*p*-Value
Time metrics			
Onset time (min)	13.22 ± 1.70	15.12 ± 2.07*	0.001
Procedure time (min)	18.12 ± 3.32	18.42 ± 3.00	0.566
Time to fully alert (min)	2.52 ± 1.11	3.62 ± 1.28*	0.001
Time to hospital discharge (min)	18.58 ± 2.98	21.21 ± 3.60*	0.001
Intubating conditions, n (%)			0.945
Excellent	22 (30.14%)	23 (31.51%)	
Good	42 (57.53%)	40 (54.79%)	
Poor	9 (12.33%)	10 (13.70%)	
Lowest oxygen saturation (%)	88.22 ± 2.16	89.90 ± 2.03	0.061
Severity of coughing, n (0/1/2/3)	2/13/44/14	4/15/36/18	0.572
Incidence of dreaming, n (%)	26 (35.62%)	30 (41.10%)	0.496
Satisfaction of patients	8.82 ± 0.71	8.66 ± 0.63	0.142
Satisfaction of bronchoscopist	9.00 (9.00–9.00)	9.00 (8.00–9.00)	0.072
Consumption of fentanyl (µg)	79.16 ± 17.20	81.26 ± 22.21	0.525
Consumption of remifentanil (µg)	134.45 ± 21.15	134.73 ± 19.74	0.934
Consumption of remimazolam tosilate (mg)	19.71 (18.36–21.29)	—	
Consumption of dexmedetomidine (µg)	—	43.15 (40.12–45.24)	
Number of remedies lidocaine, n (%)	14 (19.18%)	14 (19.18%)	1.000
Number of remedies midazolam, n (%)	26 (35.62%)	25 (34.25%)	0.863
Number of remedies propofol, n (%)	4 (5.48%)	6 (8.22%)	0.745
Number of vasoactive drugs, n (%)	16 (21.92%)	20 (27.40%)	0.565
Willing to the repeat bronchoscopy, n (%)	60 (82.19%)	58 (79.45%)	0.834

Variables presented as mean ± SD, median (interquartile range) or number of patients n (%).*p < 0.05 vs. Group RR.

Both intubating conditions and clinically acceptable intubating conditions (excellent and good intubating conditions) were similar between the two groups (*p* = 0.945, Table 3). There were also no significant differences between the two groups with respect to consumption of fentanyl and RF, number of remedies such as lidocaine, midazolam, propofol, and vasoactive drugs (*p* > 0.05; Table 3). Although the satisfaction of both patients and bronchoscopist were higher in the RR group, this difference was not statistically significant (*p* > 0.05; Table 3). Severity of coughing and patients’ willingness to repeat the bronchoscopy with the same anesthesia scheme were similar between the two groups (*p* > 0.05; Table 3).

### Safety Outcomes

**FIGURE 2:**
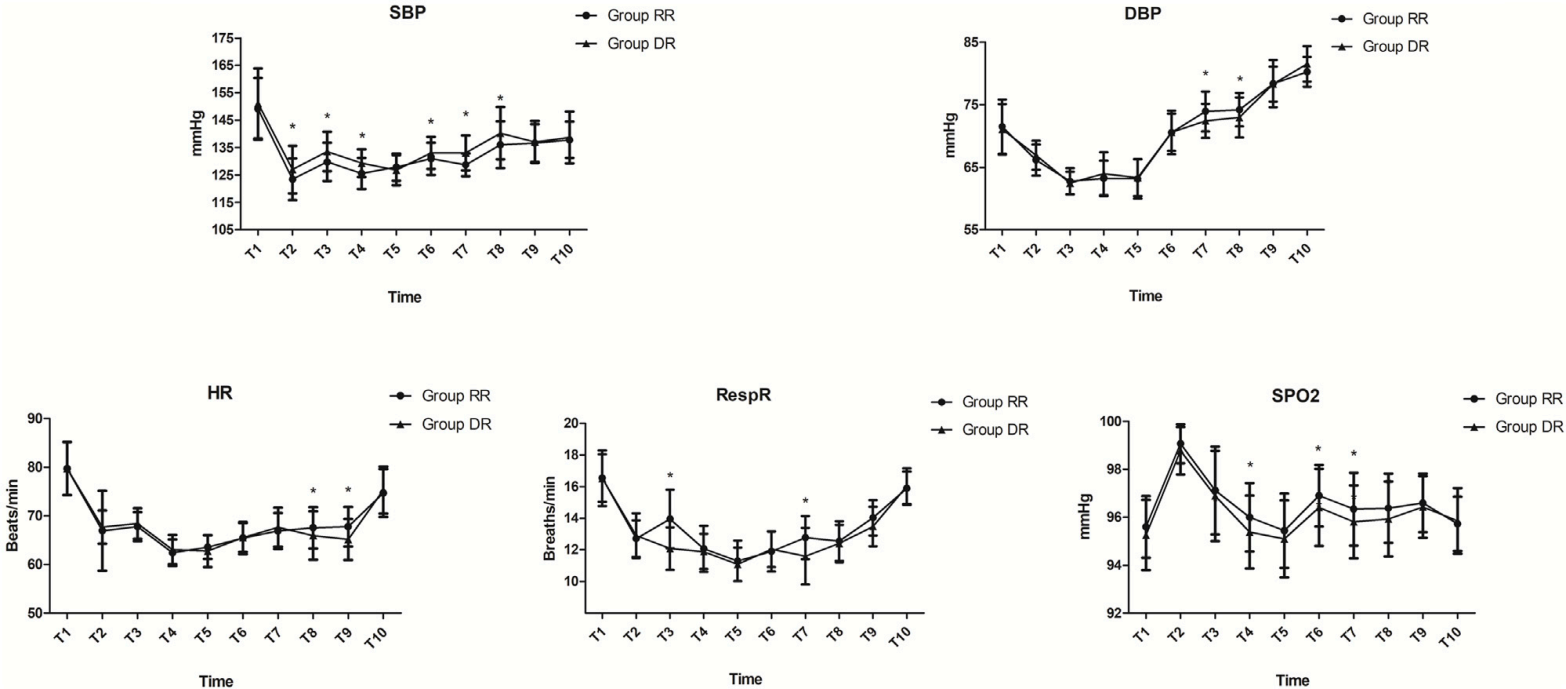
Hemodynamic measurements. In the RR group, SBP was significantly decreased from T2 to T8 except at T5, while DBP was significantly increased at T7 and T8 (*p* < 0.05). RespR was significantly increased at T3 and T7, while HR was significantly increased at T8 and T9 in the RR group (*p* < 0.05). SpO2 was significantly increased from T4 to T7 except at T5 in the RR group (*p* < 0.05). Abbreviations DBP, diastolic blood pressure; HR, heart rate; RespR, respiratory rate; RR, remimazolam tosilate-remifentanil (RT-RF); SBP, systolic blood pressure; SpO2, oxygen saturation. Time metrics are as follows: T2, immediately before start of procedure; T3, 3 min after start of procedure; T4, 6 min after start of procedure; T5, 9 min after start of procedure; T6, end of procedure; T7, 3 min after procedure; T8, 6 min after procedure; T9, 9 min after procedure

Compared with patients in the DR group, systolic blood pressure (SBP) was significantly decreased from T2 to T8 except T5, while diastolic blood pressure (DBP) was significantly increased at T7 and T8 in the RR group (*p* < 0.05; Figure 2). Similarly, the respiratory rate was significantly increased at T3 and T7, while HR was significantly increased at T8 and T9 in the RR group (*p* < 0.05; Figure 2). Compared with patients in the DR group, SpO_2_ was significantly increased from T4 to T7 except T5 in the RR group (*p* < 0.05; Figure 2). However, the lowest oxygen saturation was similar between the two groups (*p* = 0.061; Table 3).

There was no significant difference between the two groups with respect to hemodynamic adverse events (*p* > 0.05; Table 4). Eight patients in the RR group and 10 patients in the DR group had hypoxia and needed increased oxygen delivery, while 2 patients in each group required jaw thrust maneuver. However, no patients required removal of the bronchoscope and use of face mask or manual ventilation in this study. 15 patients showed arrhythmia without hemodynamic instability. Epistaxis was recorded in 3 patients (2 patients in the RR group and 1 patient in the DR group). The severity of nearly all of the adverse events was grade 1 in both groups except for 3 patients (Table 4).

**TABLE 4:** Adverse events.

Variable, n (%)	Group RR (*n* = 73)	Group DR (*n* = 73)	*p*-Value
PONV (0/1/2/3)	27/38/6/2	21/40/7/5	0.561
Hypotension	9 (12.33%)	8 (10.96%)	1.000
Hypertension	2 (2.74%)	3 (4.11%)	1.000
Bradycardia	3 (4.11%)	2 (2.74%)	1.000
Hypoxia	8 (10.96%)	10 (13.70%)	0.802
Respiratory depression	2 (2.74%)	2 (2.74%)	1.000
Arrhythmia	8 (10.96%)	7 (9.59%)	1.000
Epistaxis	2 (2.74%)	1 (1.37%)	1.000
Severity of adverse events			0.600
Grade 1	39 (53.42%)	45 (61.64%)	
Grade 2	2 (2.74%)	1 (1.37%)	
Grade 3	0	0	
Grade 4	0	0	

Variables presented as number of patients n (%). PONV, postoperative nausea and vomiting.

## Discussion

In this study, we found that RT-RF was considered non-inferior to DEX-RF in outpatients undergoing FB. The onset time, time to fully alert, and hospital discharge were all significantly shorter in the RR group, and hemodynamics were more stable in the RR group. However, intubating conditions, clinically acceptable intubating conditions, lowest oxygen saturation, coughing severity, consumption of fentanyl and RF, number of remedies, patient and bronchoscopist satisfactions, willingness to repeat bronchoscopy, and adverse events were all similar between the groups.

Guidelines from both the American College of Chest Physicians and British Thoracic Society recommend using topical anesthesia, analgesia, and sedation in all patients undergoing FB when there is no contraindication (Wahidi et al., 2011; Du Rand et al., 2013). However, anodynia fiberoptic bronchoscopy represents a challenge for anesthesiologists because they must share the same airway with the endoscopist (Semmelmann and Loop, 2022). In addition, the interactions and synergism between sedatives and opioids can significantly increase respiratory depression (Finlay and Leslie, 2021). Therefore, it is vital to maintain oxygenation via the open, flexible bronchoscope, which may also depend to a significant degree on staff expertise and equipment availability. The Australian and New Zealand College of Anesthetists guidelines recommend that sedatives and analgesics should be used at the minimum dose required for patient comfort, especially for patients with higher risk of respiratory complications such as those with COPD (Saxon et al., 2018). We excluded patients with COPD, respiratory failure, and asthma from our study based on consideration for the lack of clinical experience of RT and the safety of outpatients, even though these cases are often encountered in FB.

The benefits of premedication with anticholinergic agents during FB include drying of secretions and protection against vasovagal reaction and bronchospasm. However, no anticholinergic premedication was administered in this study, because its benefits have not been shown to be sustained through the post-bronchoscopy period (Malik et al., 2009). The use of nebulization is recommended before the procedure to both clear secretions and improve the bronchoscopic view (Muller et al., 2018). As a result, airway nebulization with 10 ml of 1% lidocaine was used for all outpatients. Topical airway anesthesia was also used in all patients to abate airway reflexes and reduce the consumption of sedation, although the advantages of sedation without oropharyngeal anesthesia for ultrathin bronchoscopy through nasal intubation has been indicated by some (Madan et al., 2021). Lidocaine is the most commonly suggested topical anesthetic, used for its wide therapeutic safety margin, short half-life, and minimal risk for toxicity. Moreover, it has multiple routes of administration, such as aerosol spray, nebulization, transcricoid or transtracheal injection, local nerve block, or “spray-as-you-go technique”. Lidocaine can also decrease ion transport across neuronal membranes, block nerve impulse conduction, and suppress coughing (Dhooria et al., 2020; Yuksel et al., 2021). We adopted the “spray-as-you-go-technique” during FB for this study taking into account acceptance, noninvasiveness, and patient comfort. The cardiac and neurologic toxicities of lidocaine are dose-related and can occur if the total topical dose exceeds 7 mg/kg or serum level exceeds 5 mg/L. (Wahidi et al., 2011) As a result, the total dose of lidocaine never exceeded 5 mg/kg in our study. Moreover, we chose to use a 1% lidocaine solution in this study to enhance patient safety, since it was reported that 1% and 2% solutions of topical lidocaine had similar efficacy (Madan et al., 2018). Partly due to the perfect topical anesthesia, the successful completion rate of the procedure in both groups was higher than previously reported (Ishiwata et al., 2018). Premedication with nebulized Dex-lidocaine inhalation could provide better operating conditions for FB; however, a variety of commonly experienced negative sensations have been recorded, such as choking and inability to swallow, increased rate of positive diagnoses, which led to several re-examinations (Gu et al., 2019).

The ideal anesthesia scheme for FB includes rapid onset and complete offset without environmental pollution of anesthetic agents; patient responsive to verbal commands; adequate spontaneous ventilation; and normal cardiovascular function. TIVA is more useful than volatile anesthetics during FB, among which benzodiazepines combined with opiates are suggested because of their synergistic effect (de Lima et al., 2018). We chose RT for this study due to its high affinity and selective ligand site on the GABA receptor, quick onset of action, rapid peak effect, relatively short duration of effect, and availability of an effective reversal agent (Antonik et al., 2012). Furthermore, despite the fact that Dex has shown superiority over midazolam in critically ill patients, there is no direct clinical evidence of superiority between Dex and remimazolam for outpatients undergoing FB (Shehabi et al., 2019). Although in the European Union (EU) remimazolam is approved for procedural sedation in adults regardless of the duration of sedation, we still excluded procedure times longer than 30 min because in the United States (United States) remimazolam is approved in adults for the induction and maintenance of procedural sedation lasting 30 min or less (Acacia Pharma Inc, 2021; European Medicines Agency, 2021). Patients with abnormalities of liver function were also excluded, since the pharmacokinetics of RT is altered in patients with severe hepatic impairment (Stohr et al., 2021). In both the EU and United States, an initial remimazolam dose of 5 mg is recommended for the induction of procedural sedation, followed by a 2.5 mg maintenance dose, though the tosylate salt of remimazolam is not approved in both EU and United States. However, a recent study shows that the ED_95_ of RT was 0.219 mg/kg in the Chinese population; therefore, we administered RT at 12 mg/kg/h during the induction of procedural sedation for 10 min according to that study (Chen W et al., 2021).

Dex is an α-adrenergic agonist that decreases the plasma concentration of catecholamines in both the brain and spinal cord to provide sedation, analgesia, and loss of anxiety. Dex also reduces tracheal ring contractions and acetylcholine release. As a result, it has recently been used during FB (Zhang et al., 2021). In our study, we found that the onset time, time to fully alert, and hospital discharge were all significantly longer in the DR group, although the intubating conditions were similar, which may be due to the longer onset and offset sedation activity and smaller dosage of Dex used in this study. Besides, the recovery time in the RR group was shorter than in previous studies, which may result in reduced costs and increased utilization of procedure and recovery rooms (Lee et al., 2019). It has been reported that dreaming can be produced even with brief surgery. Compared to midazolam, pre-injection of Dex before FB significantly decreased the incidence of dreaming. The reason may be partly because both locus ceruleus and dorsal raphe nuclei play critical roles in the regulation of sleep, which was the action site of Dex (Chen L et al., 2021). However, we did not record this difference in this study.

The control of coughing by opioids may be outweighing the risk for hypoventilation and apnea (Tschiedel et al., 2021). Because of the disadvantages of fentanyl in terms of offset time, hepatic metabolism, and accumulation and prolonged effect with continuous infusion, we chose RF as the preferred opioid in this study (Lin et al., 2019). Dex combined with propofol (10–20 mg) has been shown to suppress coughing, and is an appropriate choice for rigid bronchoscopy in malignant airway fistula (Kowalski et al., 2020). However, we did not record any difference between the two groups with respect to both intubating conditions and clinically acceptable intubating conditions, which may be due to the different degrees of stimulation between FB and rigid bronchoscopy. Propofol is increasing in popularity because of its amnestic properties, with a quicker onset, faster recovery time, and improved procedure tolerance. As a result, we chose propofol as the remedial drug for this study. The dose of propofol, other than RF infusion, tends to decrease over time during FB, and as a result, it should be cautioned that severe respiratory depression may occurr (Park et al., 2020). In addition, both RF and propofol can cause hypotension and bradycardia, thus when used in combination they could have a synergistic effect on hemodynamics. For this reason, only 10–30 mg propofol were used as the final remedial medication in our study.

Consistent with the results of previous studies, hypoxia was one of the most common complications during FB in our study (Bhat et al., 2019). Less frequent episodes of hypoxemia and severe hypoxemic events, as well as higher mean lowest SpO_2_ values, have been shown when capnography is used, due to improved monitoring, especially in sedated patients receiving supplemental oxygen (Parker et al., 2018). As a result, PetCO_2_, a noninvasive and continuous method to monitor ventilation, is expected to be a useful monitoring device for non-intubated patients during FB and was used for all outpatients in this study according to the recommendation of the Association of Anaesthetists of Great Britain and Ireland. However, end tidal CO_2_ measurements were considered unreliable in a prior study where most patients showed false low-end tidal CO_2_ levels due to an unsealed dual nasal cannula and room-air dilution of expired gases (Ibrahim et al., 2019). Despite the fact that recent evidence supports the use of capnography in deep sedation, its use in moderate sedation has not been shown to improve patient safety (Vakil et al., 2018). Despite various monitoring methods, the incidence of oxygen desaturation in this study was still relatively high, which may be due to inhibition of the respiratory centre in the brainstem by RF, especially when associated with benzodiazepines; hypoventilation secondary to sedation; and airway obstruction by the bronchoscope itself (Zha et al., 2021).

There was no significant difference in adverse events between the two groups in our study. Compared with the results of a previous study, the incidence of postoperative nausea and vomiting was significantly reduced in our study, which may partly be due to the fact that dexamethasone decreased postoperative airway inflammation, swelling, pain, and discomfort (Feng et al., 2022). Remimazolam has been associated with an increase in HR and a mild increase in QT prolongation. As a result, the United States Food and Drug Administration warns that remimazolam must be used in the presence of personnel and equipment for monitoring and resuscitation, especially if used concomitantly with opioid analgesics and other sedative hypnotics (Pantos et al., 2021). However, no patients experienced serious adverse events in this study. Bradycardia and hypotension were the most common adverse reactions in the Dex group, especially before commencing FB. However, we reduced the loading dose and slowed the infusion, which might have reduced hemodynamic fluctuations. The incidence of hypotension was higher than in previous studies, which may be due to the large doses of RF used in our study (Dhooria et al., 2020).

This study has some limitations and the outcome may be influenced by the following factors. First, we only used a subjective evaluation of the level of sedation, which could disturb the procedure. Objective criteria to measure the level of sedation, such as the bispectral index (BIS), electroencephalogram, and auditory evoked potentials, may be more predictive of the level of sedation, although the correlation of BIS was weaker for benzodiazepines, such as midazolam and remimazolam (Punjasawadwong et al., 2018). Second, the optimal concentration of RT combined with RF should be further determined. Third, we only included outpatients with ASA I-II, more studies are needed to verify the results of this study especially for special patients. Finally, the outpatient population came from a tertiary referral center, which may limit the generalizability of the results of this study.

## Conclusion

We found that RT-RF has non-inferior efficacy, better time metrics and hemodynamic stability than Dex-RF, which suggests that RT-RF is more suitable for outpatients undergoing FB.

## Data Availability

The data analyzed in this study is subject to the following licenses/restrictions: The datasets generated during and/or analyzed during the current study are not publicly available due to the privacy policy but are available from the corresponding authors upon reasonable request. Requests to access these datasets should be directed to QL, 13869530842@163.com.
